# Big Data in Oncology Nursing Research: State of the Science

**DOI:** 10.1016/j.soncn.2023.151428

**Published:** 2023-04-19

**Authors:** Carolyn S. Harris, Rachel A. Pozzar, Yvette Conley, Manuela Eicher, Marilyn J. Hammer, Kord M. Kober, Christine Miaskowski, Sara Colomer-Lahiguera

**Affiliations:** a Postdoctoral Scholar, School of Nursing, University of Pittsburgh, Pittsburgh, Pennsylvania, USA; b Nurse Scientist at Phyllis F. Cantor Center for Research in Nursing and Patient Care Services, Dana-Farber Cancer Institute, Boston, Massachusetts, USA and Instructor at Harvard Medical School, Boston, Massachusetts, USA; c Professor, School of Nursing, University of Pittsburgh, Pittsburgh, Pennsylvania, USA; d Associate Professor and Director of the Institute of Higher Education and Research in Healthcare (IUFRS), Faculty of Biology and Medicine, University of Lausanne, and Lausanne University Hospital, Lausanne, Switzerland; e Director, The Phyllis F. Cantor Center for Research in Nursing and Patient Care Services, Dana-Farber Cancer Institute, Boston, Massachusetts, USA and Lecturer at Harvard Medical School, Boston, Massachusetts, USA; f Associate Professor, School of Nursing, University of California, San Francisco, California, USA; g Professor, Schools of Medicine and Nursing, University of California, San Francisco, California, USA; h Senior Nurse Scientist and Junior Lecturer, Institute of Higher Education and Research in Healthcare (IUFRS), Faculty of Biology and Medicine, University of Lausanne, and Lausanne University Hospital, Lausanne, Switzerland

**Keywords:** Big data, Data science, Malignant neoplasms, Nursing research, Oncology nursing

## Abstract

**Objective:**

To review the state of oncology nursing science as it pertains to big data. The authors aim to define and characterize big data, describe key considerations for accessing and analyzing big data, provide examples of analyses of big data in oncology nursing science, and highlight ethical considerations related to the collection and analysis of big data.

**Data Sources:**

Peer-reviewed articles published by investigators specializing in oncology, nursing, and related disciplines.

**Conclusion:**

Big data is defined as data that are high in volume, velocity, and variety. To date, oncology nurse scientists have used big data to predict patient outcomes from clinician notes, identify distinct symptom phenotypes, and identify predictors of chemotherapy toxicity, among other applications. Although the emergence of big data and advances in computational methods provide new and exciting opportunities to advance oncology nursing science, several challenges are associated with accessing and using big data. Data security, research participant privacy, and the underrepresentation of minoritized individuals in big data are important concerns.

**Implications for Nursing Practice:**

With their unique focus on the interplay between the whole person, the environment, and health, nurses bring an indispensable perspective to the interpretation and application of big data research findings. Given the increasing ubiquity of passive data collection, all nurses should be taught the definition, characteristics, applications, and limitations of big data. Nurses who are trained in big data and advanced computational methods will be poised to contribute to guidelines and policies that preserve the rights of human research participants.

## Introduction

With advances in technology, the conceptualization, definition, and use of big data in research have evolved. An early definition of big data included three main attributes, known as the three Vs: “high volume, high velocity, and/or high variety information assets that demand cost-effective, innovative forms of information processing that enable enhanced insight, decision making, and process automation.” ^1^ Volume refers to a large amount of data; velocity refers to a high-frequency stream of incoming data; and variety refers to a wide range of data sources or types that require different syntactic formats. Additional Vs that were added over time include variability (ie, the extent to which investigators must differentiate “noise” from important data), veracity (ie, data quality or accuracy), and value (ie, the importance of the data).^2–4^

Big data in health care encompasses large amounts and diverse types of data from the rapid and increased digitization of individual patient information. The use of big data to improve health outcomes requires cost-effective collection of information from different sources, conversion and storage of data into specific formats, and processing and analyses of this information according to the needs of the user.^5^ Data can be obtained from internal or external sources, including clinical and biological data from electronic health records (EHRs) or research (eg, omics), public or government records (eg, public datasets), or financial records (eg, insurance or payor).^6^ In addition, big data includes patient-generated health data (PGHD). PGHD are “health-related data – including health history, symptoms, biometric data, treatment history, lifestyle choices, and other information – created, recorded, or gathered by or from patients (or family members or other caregivers) to help address a health concern.”^7^ Social media can be a complementary source of health-related data and may be used for epidemiological surveillance or control.^8^

The use of high-volume datasets in nursing research is well established.^9^ For decades, nurse scientists have led analyses of data collected as part of routine health care and administration. Several landmark nursing studies have leveraged clinical and administrative claims data to inform safe staffing ratios^10^ and approaches to pressure ulcer^11^ and fall risk assessment.^12^ Leveraging routinely collected data offers an alternative to the collection of large quantities of data directly from research participants, which may impose a burden on some individuals with a health impairment.^13^ When large datasets include nurse-sensitive indicators (eg, patient falls, nosocomial infection rates), analyses may provide evidence for the value of nursing care and its association with health outcomes.^14^

Over the years, advances in computing power and computational methods (see Papachristou et al in this Big Data Special Issue) have expanded the potential for high-velocity and high-variety data to meaningfully inform patient care.^15^ Oncology nurses tailor their interventions to account for the biological, social, cultural, and environmental factors that may affect a person’s well-being. High-variety data have the potential to inform this patient-centered approach. For example, big data often underlies precision health initiatives that aim to deliver health care that is optimized for a person’s unique genetic or genomic composition, lifestyle influences, and the context in which they live.^16^ Large datasets composed of information from a variety of sources can help oncology nurse scientists identify novel biological, psychosocial, or environmental factors that predict or contribute to disease burden. In addition, analyses of big data may support clinical decision making by identifying complex combinations of factors that predict adverse health outcomes. In turn, these analyses may allow nurses to identify patients who may benefit from proactive interventions.^13^ The authors aim to describe some of the most common sources of big data available to oncology nurse researchers, describe access considerations to these data sources; and provide exemplars of big data research from oncology nurse scientists. In addition, the authors describe important ethical issues that need to be considered when amassing, using, and reporting findings from big data analyses and suggest directions for future research.

## Sources of Big Data and Access Considerations

### Electronic Health Record

The EHR exemplifies big data. It consists of a large volume of clinically relevant information that is continually updated and derived from a variety of sources. Data stored in the EHR are varied and may include clinician notes, vital signs, laboratory reports, telemetry data, imaging data, ICD codes, and PGHD (eg, symptom reports). Investigators can extract structured data from the EHR to characterize study participants. Structured data have a standardized format and are easily stored in an organized database. Examples of structured data that are relevant to oncology nursing research include date of cancer diagnosis, blood pressure, and tumor stage. Conversely, unstructured data lack a standardized format and are more difficult to organize. Examples of unstructured data include clinician’s narratives, scanned handwritten notes or test results, and free-text findings from imaging studies. Because manual review and extraction of unstructured data are time-consuming and costly,^17^ these data are currently underused in research. The underrepresentation of unstructured data in the oncology literature represents a missed opportunity, given that an estimated 70% to 80% of EHR data are unstructured.^18^

Novel computational methods have the potential to analyze large volumes of unstructured EHR data efficiently and accurately. For example, in patients with multiple chronic conditions, natural language processing (NLP) was used to analyze and extract symptom data from nursing notes to identify groups of patients with similar symptom cluster profiles.^19,20^ In the oncology setting, NLP was used to analyze narrative EHR data from 808 patients receiving palliative care at the end of life.^17^ The investigators sought to develop and evaluate models to detect social distress, spiritual pain, and severe symptoms from 1,554,736 clinician narratives. The investigators developed core search terms for each construct, trained NLP models by manually annotating the presence or absence of each construct in a subset of the data, and evaluated each model’s performance with the remaining data. Although the NLP models for detecting social distress, spiritual pain, severe pain, dyspnea, and nausea demonstrated high accuracy, those for detecting severe insomnia and anxiety demonstrated moderate accuracy. Although the investigators found that the positive predictive values of the NLP models for detecting social distress and spiritual pain were poor, this finding may reflect the quality of the data recorded. One adage that applies to big data analyses is “garbage in, garbage out,” which refers to the importance of training computational models on high-quality data. Nevertheless, NLP is approximately 10 times faster than manual coding and may identify information that human analysts overlook.^21^ The development and refinement of additional computational methods in coordination with efforts to promote standardization in clinical documentation will facilitate oncology nurse scientists’ ability to leverage unstructured EHR data.

### Patient-Generated Health Data and Remote Monitoring

Technological advancements have enabled more powerful and portable personal electronic devices that consumers can wear and/or interact with, producing vast amounts of data. Smartphones, mobile health applications (apps), and wearable devices have increased the frequency, amount, and types of PGHD available. In contrast to clinical data, PGHD allow patients to be responsible for capturing, recording, and deciding whether and with whom to share their data.^7^

PGHD allows a continuous tracing of consumer-specific entries, such as those related to location, physical activity, heart rate, blood pressure, glucose, temperature, sleep patterns, or adherence to medication, among others. Remote longitudinal and real-time monitoring can standardize the collection of data across patients and clinics and decrease information gaps (eg, recent changes in a patient’s condition; symptoms that prompt a change in the care plan).^22^ In addition, remote digital methods may facilitate retention of and access to a wider and more diverse group of participants, reducing costs and time to create targeted cohort groups, in comparison to traditional clinical studies.^23,24^ Furthermore, PGHD may offer cost-effective strategies by optimizing cancer care outside of the clinic.^25^ In clinical research, detailed information about the time of collection, amount, or combination of data sources can help to standardize and capture more precise and frequent data to understand mechanisms and toxicities of cancer treatments and improve the efficiency of oncology clinical trials.^26,27^ Moreover, predictive models of disease states can be tested and health-promotion interventions created. The use of PGHD enables a shift from provider-driven to patient-led activities that enables self-monitoring and self-management and fosters patient engagement.^28^ However, additional research is needed on the legal, ethical, feasibility, and modeling issues related to the acquisition and use of PGHD.

### Wearable Health Devices (passive reporting)

A wearable is a device with a sensor that can collect health-related data remotely with the advantage of minimizing discomfort and interference with normal human activities. This approach makes it possible to monitor patients in their own environment.^29^ Wearable and remote patient monitoring devices may be fastened to the wrist, upper arm, waist, hip, or other body parts. These devices can provide biometric data, including heart rate, electrocardiogram, respiratory rate, blood oxygen saturation, blood glucose, sleep pattern, and body temperature. The collection of data from wearable and remote patient-monitoring devices can take place in real time or during scheduled data transfers. In this sense, these devices combine the three main Vs of big data: large amounts of data (volume) that are collected in real time or at high frequency (velocity) from a wide range of data sources (variety).

In the oncology setting, several examples exist of the use of wearable and remote patient-monitoring devices to improve patient outcomes during and after cancer treatment. As part of a European project titled Integrated Network for Completely Assisted Senior Citizens’ Autonomy (inCASA),^30^ a home-based platform was used to monitor real-time symptoms in patients receiving chronomodulated chemotherapy at home.^31^ Circadian rest-activity rhythm and sleep were measured with a wrist accelerometer, body weight changes with a dedicated scale, and symptom information with a questionnaire completed on an interactive electronic screen. Evidence for the acceptability of this approach included 5,891 data points collected over 364 patient-days out of the 8,736 expected (67.4%), with a median daily adherence of 73%. This approach allowed a day-to-day multidimensional and accurate evaluation of each patient’s response to the treatment and helped document the safety of chronomodulated triplet chemotherapy delivery in the patient’s home.

In contrast, other studies reported suboptimal adherence to wearable health devices. The OncoWatch 1.0 study investigated the feasibility of using smartwatches to monitor heart rate and physical activity in patients with head and neck cancer who were receiving radiotherapy.^32^ Only 31% of patients adhered to the study protocol that entailed wearing a smartwatch for 12 hours per day during and for 2 weeks after radiotherapy. The investigators proposed that the task of charging the watch and not being able to use the watch for personal purposes led to low adherence.

Another example of the use of sensors for home-based cancer symptom management is Behavioral and Environmental Sensing and Intervention for Cancer (BESI-C).^33^ In this study, dyads of patients with cancer and their primary caregivers were followed to monitor cancer pain and distress at home. Environmental sensors assessed the home context (eg, light and temperature), and Bluetooth beacons located dyad positions. Both patients and caregivers wore smart-watches to record and characterize pain events. This study introduced a new approach to monitoring and mitigating the escalation of cancer pain and distress by controlling environmental and contextual factors at home. Participants reported that the intervention was meaningful and not burdensome.

### Patient-reported data (active reporting)

Patient-reported outcomes (PROs) are systematic ways of measuring patients’ subjective views about the impact of their disease and its treatment. From a value-based care point of view, collecting PRO data could help to evaluate, monitor, and improve provider and setting performance, or establish standards and benchmarks to measure the effectiveness of a health system.^34^ One study^35^ identified three potential uses of “Big PRO” data: (1) to guide individual care through real-time monitoring; (2) to develop population-level prognostic models to predict patients most likely to benefit from an intervention and to identify those who are a priority for care; and (3) to enrich observational research in real-world trials. Despite their established use in clinical trials, PROs are not universally collected in real-world clinical settings. One barrier to the integration of PROs into routine care is that many EHRs are not designed to meaningfully display and assist clinicians to interpret PRO data.^36,37^ For nursing, the lack of PROs in EHRs limits the extent to which nursing interventions such as patient education, symptom evaluation, and symptom management can be measured and evaluated.^38^

In 2013, the Patient-Centered Outcomes Research Institute (PCORI) in the United States launched PCORnet, the National Patient-Centered Clinical Research Network, a major initiative to create an effective and sustainable infrastructure to support researchers in learning from clinical and patient-reported outcomes in large observational studies.^39^ Another example is the Dutch population-based Patient-Reported Outcomes Following Initial treatment and LongTerm Evaluation of Survivorship (PROFILES) registry, which combines longitudinal PRO measures, objective measures, and cancer registry, ambulatory, and pharmacy data.^40^

In France, the CANcer TOxicities (CANTO) longitudinal cohort study (NCT01993498) is developing a database of chronic treatment-related toxicities in 14,750 women with stage I to III breast cancer.^41^ The aims of the study are to quantify the impact of treatment toxicities and to generate predictors of chronic toxicity in patients with nonmetastatic breast cancer. CANTO collects PROs (ie, quality of life, psychological, behavioral), as well as clinical, treatment, toxicity, socioeconomic, and biologic data. These initiatives will allow the full integration of PROs and information related to their impact into EHRs, claims databases, and other sources of big health data. In addition, initiatives such those undertaken by the Organization for Economic Cooperation and Development^42^ and the International Consortium for Health Outcomes Measurement^43^ aim to support and develop a coherent and comprehensive approach to standardizing and implementing the systematic collection of PRO data internationally.

### Large Public Datasets

A major challenge faced by researchers is the acquisition of high-quality data. Prospective data collection can be an expensive process that is time intensive for both researchers and patients. Due to funding constraints, researchers must make difficult decisions about what types of data to collect, number of assessments, and number of patients. Furthermore, multiple years pass between the grant writing process and the beginning of data analysis, which impedes progress in oncology research. The availability of publicly available datasets with large samples (eg, >1000 participants) that acquire data longitudinally and include various types of data (eg, symptom severity, gene expression) can accelerate oncology research. To improve the management of data produced by studies funded by the National Institutes of Health (NIH) in the United States and increase the responsible sharing of these data, the Policy for Data Management and Sharing^44^ was enacted requiring that researchers of NIH-funded studies share their data with a quality data repository (eg, Database of Genotypes and Phenotypes [dbGaP]). Given that this policy went into effect as of January 2023, data within publicly accessible data repositories will expand in volume exponentially. In addition to the databases previously described (ie, PROFILES, CANTO), the next section of this paper describes five publicly available datasets with a high variety, velocity, and volume of data that oncology nurse scientists can access to explore a variety of research questions.

### National biobanks

The United Kingdom (UK) Biobank is a biomedical database composed of growing volumes of a variety of data used to identify the underlying causes (eg, environmental, genetic) of various diseases. Recruitment of more than 500,000 UK citizens took place between 2006 and 2010, and the study continues to prospectively collect data on all living participants.^45^ The target age for recruitment was 40 to 69 years because this age period is associated with increased development of various conditions, including cancer. Participants were required to be registered with the universal health care system of the UK, provide consent for long-term follow-up, and allow for study access to their health records. Therefore, detailed health records on cancer and death registry data and inpatient and primary care records are updated annually and are available on all participants.^46^ Data available for analyses include detailed questionnaires on health, lifestyle, and exposures; physical measures and accelerometer data; whole genome and exome sequencing on all participants; blood, urine, and saliva for proteomic, metabolomic, and telomere analyses; and magnetic resonance imaging of the brain, heart, and full body. Access to this rich resource is available to the international scientific community through application.

The NIH launched the All of Us Research Program in 2015, recognizing that a “one size fits all” policy for disease prevention and treatment may not be effective for every person.^47^ All of Us proposes that to determine the specific risk factors for various diseases and to develop individualized treatments, the influence of one’s environment, lifestyle, family history, and genetic makeup on disease development and treatment efficacy must be evaluated. Acknowledging the historic absence and exclusion of people from racial and ethnic minority communities, rural communities, and lower socioeconomic status in biomedical research,^48^ All of Us is committed to the recruitment of participants who reflect the diversity of the United States. To date, All of Us is more than halfway to its goal of recruiting 1 million participants and plans to collect additional data over time. Types of data being collected include patient-reported surveys on one’s environment, lifestyle, and other social determinants of health; EHR data; physical measures; blood, urine, and saliva samples; and digital health data. While recruitment and data collection are ongoing, current deidentified data can be accessed on three tiers: Public Tier (ie, view data snapshots, no registration required), Registered Tier (ie, includes data from EHRs and surveys, registration required), and Controlled Tier (ie, genomic data, registration and prior approval required).

Using cross-sectional data from 14,127 participants in the All of Us Research Program, symptom phenotypes in participants diagnosed with one or more chronic conditions (ie, cancer, chronic obstructive pulmonary disease, heart failure, and/or type 2 diabetes mellitus) and risk factors that predicted membership in these symptom phenotype groups were evaluated.^49^ Cohort Builder within the All of Us Researcher Workbench was used to identify study participants for analysis. Eligible participants were required to have one or more of the prespecified chronic conditions and complete response data on fatigue, emotional distress, and pain items on the Overall Health Survey that was collected after diagnosis. Using hierarchical cluster analysis, four distinct symptom phenotypes were identified (ie, mild symptoms, severe emotional distress, severe pain, severe symptoms). Participants who forwent or delayed medical care or rated their mental or physical health as poor were more likely to belong to the severe emotional distress, pain, or symptom phenotypes.

### National survey data

Another type of large, publicly available data that researchers can use is data compiled from national or international surveys. For example, in an effort to improve patient-centered care, health care systems and governments are increasingly using large-scale, population-wide, patient-reported surveys to examine patients’ experiences across the cancer-care continuum. These surveys provide a perspective on the aspects of cancer care that patients find most important. Notably, patient-reported experiences complement data on health outcomes (eg, treatment effectiveness, mortality), which together provide a more holistic picture of the quality of health care.^50^

In the United States, the Consumer Assessment of Healthcare Providers and Systems Cancer Care Survey examines patient experiences in the context of their interactions with various clinicians and staff (eg, communication, perceived respectfulness of staff), experiences with health care facilities (eg, care coordination, timeliness of appointments), and perception of overall cancer care.^51^ Using a similar survey, the UK uses the Cancer Patient Experience Survey to assess changes in cancer care and as a tool to inform quality improvement.^52^ The Patient-Reported Indicator Survey (PaRIS) of People Living with Chronic Conditions measures both patient-reported experiences (eg, care coordination, wait times) and PROs (eg, quality of life, physical functioning) in adults living with one or more chronic conditions.^42^ Because PaRIS is an international survey, researchers and institutions can compare data within and across countries. Researchers have used data generated from large-scale patient-experience surveys to examine factors associated with patient care experiences in older patients with hematologic malignancies,^53^ associations between having a better care experience with a clinical nurse specialist and overall survival in patients with heterogeneous types of cancer,^54^ and variations in patient experiences with cancer care by type of cancer in patients with heterogeneous types of cancer.^55^

### Social Media

Worldwide, an estimated 4.74 billion people use social media.^56^ Social media platforms allow users to engage with each other and share user-generated content.^57^ The most widely used social media platforms include YouTube, Facebook, and Twitter.^58^ Social media may be used by individuals to exchange social, emotional, and practical support related to a health condition or to find and share health information.^59^ To date, investigators have used social media to recruit research participants rather than as a source of research data. However, investigators may face several challenges related to participant misrepresentation when they use social media platforms for recruitment.^60^ Investigators who analyze content that social media users share publicly may avoid these challenges. Although user-generated social media content may shed light on the experiences of people with cancer and other conditions, the unstructured nature of this content has limited the extent to which it has been formally analyzed.

Online discussion forums represent an especially promising source of high-velocity unstructured health data. In a study that aimed to develop an automated model to classify the needs expressed by patients and caregivers online,^61^ 853 messages shared in an online health community for people with ovarian cancer and their caregivers were analyzed. First, messages that referenced physical, psychological, social, and information needs were manually annotated. Next, a machine learning model that used a “bag of words” representation was built, using the combination and frequency of the words in each message to predict the needs expressed in each message. The resultant classification model was able to identify different types of needs with a high level of accuracy. These findings suggest that novel computational methods such as machine learning are a feasible approach to use to analyze large amounts of unstructured user-generated data.

### Omics

To determine the complex mechanisms that underlie common symptoms in patients with cancer, oncology nurse scientists are increasingly incorporating omics approaches to their research. The various types of omics data can be conceptualized as levels of biological data (eg, genomics, transcriptomics, proteomics). Given that each type of omics data provides valuable and unique insights into the molecular underpinnings of various conditions, researchers may select one or more types of omics data for their analyses based on their research questions and/or hypotheses.^62^ For example, epigenomics data (eg, DNA methylation) can be used to examine linkages between social determinants of health and symptom or health outcomes.^63^ Findings from these studies have the potential to identify biomarkers of disease or symptoms and lead to the development of tailored and targeted interventions.

For example, an interdisciplinary team of oncology nurse and physician researchers, bioinformaticians, and molecular geneticists integrated a variety of high-volume data types to identify a potential target for intervention in breast cancer survivors with paclitaxel-induced peripheral neuropathy. In their first study,^64^ a transcriptome-wide differential gene expression analysis (11,487 genes) was performed between breast cancer survivors who did (n=25) and did not (n=25) develop paclitaxel-induced peripheral neuropathy as a result of paclitaxel administration. With the use of pathway impact analysis, 53 significantly perturbed pathways were identified between the survivor groups. In the second study,^65^ the authors further interrogated the hypoxia-inducible factor 1 (H1F-1) signaling pathway that was identified in their previous analysis using both transcriptomic and epigenomic data. Of the 100 genes in the H1F-1 signaling pathway, eight were found to be differentially expressed and methylated between the survivor groups. Next, these eight genes were evaluated in preclinical models of neuropathic pain using publicly available datasets from the National Center for Biotechnology Information Gene Expression Omnibus^66^ (ncbi.nlm.nih.gov/geo/). Differential expression and methylation of the mitogen-activated protein kinase I interacting serine/threonine kinase I gene was to be found associated with neuropathic pain in both breast cancer survivors with paclitaxel-induced neuropathy and preclinical models of neuropathic pain. Taken together, these findings highlight the strengths of interdisciplinary collaboration and use of multiple types of data sources (eg, omics, preclinical) and suggest a potential target for intervention.

## Skills Needed to Harness Big Data

Given that big data is increasingly being used to inform clinical practice, it is imperative that nurse scientists have the requisite knowledge and skills to use these data. All nurses should be taught the definition, characteristics, applications, and limitations of big data.^67^ Nurse scientists who intend to collect and analyze big data should pursue training in the computational methods described in Papachristou et al’s commentary on big data analytics in this Big Data Special Issue. A nonexhaustive list of educational opportunities for nurse scientists who wish to pursue training in the collection or analysis of big data is provided in Table 1. In addition, nurses in all roles should be skilled at interdisciplinary collaboration. Data scientists, bioinformaticians, and computer scientists have the expertise to support nurses to extract, organize, and analyze large datasets. In turn, nurses provide the holistic perspectives required to interpret and act on the results of these analyses to improve the well-being of individuals, families, and communities.^9^

## Ethical Considerations with Big Data

### Informed Consent

Informed consent in the context of big data is a subject of significant ethical discourse that is centered on concerns about participant autonomy.^68–70^ For example, participants who consent to have their blood collected for a genome-wide association study may not anticipate the discovery of secondary findings related to a pathogenic gene variant, such as for *BRCA1* or *BRCA2.* In addition, they may not be fully prepared to share this information with relatives or future offspring. While a participant may provide a specimen for a candidate gene association study of inflammatory markers, in a case where broad consent is obtained, this specimen may be used for future research (eg, genome-wide study of pathogenic variants). These considerations must be included in the informed consent process to ensure autonomy is upheld. For more detailed information on broad consent in the context of omics research, refer to the excellent review by Williams and Anderson.^71^

In terms of informed consent for studies using social media data, individuals grant specific permissions to social media platforms during registration. However, these permissions are not knowingly extended to recruitment and data collection for research.^72,73^ Therefore, researchers need to identify their presence in both public and private social media groups and be transparent in their intentions with potential and recruited participants. In addition, given that assurances of anonymity in social media research cannot be promised, strict procedures to strengthen confidentiality must be made throughout the research process.^73,74^

### Duty to Report or Intervene

When accruing, analyzing, or mining big data, procedures must be in place to respond to or intervene on issues of participant safety or to address incidental findings. These considerations are important given that the methods for big data collection and analysis may not facilitate the real-time evaluation of individuals’ responses. For example, in clinical trials, the collection of PRO data on emotional distress or pain may identify individuals experiencing severe levels of distress or pain that necessitate a timely response. To identify these patients in real-time, researchers can implement specific PRO thresholds that trigger an alert, identify the individual, and allow researchers or clinicians to intervene in a timely manner.^75^ In terms of omics data, secondary findings, such as pathogenic or expected pathogenic variants, may be identified.^76^ For example, findings from a study that conducted whole-exome sequencing for 49,960 participants in the UK Biobank reported that 2.7% of participants had a pathogenic or likely pathogenic variant as defined by the American College of Medical Genetics and Genomics Secondary Findings Guidelines.^77^ Under the UK Biobank informed consent, these results cannot be shared with participants or their clinicians. In the All of Us Research Program that includes an evaluation of 59 pathogenic or expected pathogenic variants, participants are given the option during the informed consent process to receive this information.^78^ In addition, if medically actionable variants are identified, participants will receive genetic counseling.

### Security and Privacy

Given the depth and breadth of big data, security of these data is a significant issue that will only magnify as data accrues. For example, data breaches in healthcare systems containing millions of EHRs are not uncommon.^79^ Nurse engagement in all steps of the research process is required to ensure that safeguards are in place to protect patient data.

Specific security and privacy concerns apply to data collected from sensors and wearable devices. When third-party technologies are used to collect research data, the amount and type of data that device manufacturers collect from participants are often beyond the investigator’s control.^80^ Both breaches in data security and increased surveillance have the potential to harm participants by violating their right to privacy. Investigators who collect data using sensors and wearable devices can support participants’ right to privacy by including information about how data may be used by third parties in the informed consent document.^80^

Engagement in policy development to ensure patient protections is an important role for oncology nurse scientists who use big data.^81^ In addition, nurse clinicians and researchers must have a keen knowledge of the policies that regulate big data and the limitations of these policies to ensure that all facets of the polices are adhered to and to serve as a resource to patients. One example of policy that seeks to regulate big data is the General Data Protection Regulation of the European Union. Effective since 2018, this law restricts how any entity, within or outside of the European Union, may handle or process personal data of citizens or residents of the European Union.^82^ Reinforced with steep fines, this law outlines the rights of the data subject (eg, right to restriction of processing), rules of consent, conditions when personal data may be processed, responsibilities of data controllers and processors, and expectations for data protection.

In terms of genetic data, the Genetic Information and Nondiscrimination Act (GINA) was passed in the United States to protect individuals who provide their genetic information for research studies from the potential for genetic discrimination in terms of employment and health insurance.^83^ Specifically, employers cannot discriminate in terms of hiring or firing an individual based on their genetic information and cannot request this information from employees. In addition, health insurers cannot deny coverage or change insurance rates based on an individual’s genetic information. Genetic information in these instances extend beyond the individual and include family members. However, GINA does not protect individuals from genetic discrimination in terms of life insurance, disability insurance, long-term care insurance, or other uses of genetic information.^84^ Furthermore, GINA only applies to individuals who have not been diagnosed with a medical condition associated with their genetic makeup. Therefore, this law does not apply to cancer survivors. Similar laws were implemented in Canada (ie, the Genetic Non-Discrimination Act)^85^ and Germany (ie, German Human Genetic Examination Act).^86^ For ongoing discussion on the ethical, legal, and social implications of genomics research, refer to the review by Hammer.^87^

### Underrepresentation in Big Data

As with other types of research, the underrepresentation of individuals from minoritized racial, ethnic, sexual, and gender groups in big data delays progress toward precision health^74^ and can lead to harmful study findings and/or interpretation. For example, in a study that examined the ancestral population diversity in two public data sources from the NIH (ie, Genome-Wide Association Study Catalog, dbGaP), African, Latin American, and Asian ancestral populations were significantly underrepresented.^88^ In genomic research, underrepresentation of these ancestral populations in diverse datasets may hinder the identification of gene–disease associations that are uncommon in European ancestral populations, lead to the identification of incorrect associations, and limit the generalizability of findings in the clinical setting.

Underrepresentation in big data is particularly problematic when these data are used to train machine learning models. For example, lack of racial and ethnic diversity in publicly available radiology datasets has limited the ability of artificial intelligence programs to correctly identify breast lesions in patients of color.^89,90^ To address this issue, a team of researchers from Emory University in the United States developed the EMory BrEast imaging Dataset (EMBED), which includes detailed demographic, lesion, and pathological data on a diverse sample of nearly 116,000 patients.^90^ The researchers hypothesize that this diverse dataset will allow for the “development and validation of deep learning models for breast cancer screening that perform equally across patient demographic characteristics and reduce disparities in health care” (p. 7).^90^ Importantly, underrepresentation is not the only source of potential bias in research that uses big data.^91,92^ Investigators have a responsibility to familiarize themselves with the principles of algorithmic fairness and the potential for latent biases to influence the results of big data studies.

## Future Directions and Conclusion

The authors summarized the state of the science of big data in oncology nursing research by describing common sources of big data, reviewing access considerations to these data sources, and providing exemplars on how these sources can be used to examine research questions relevant to oncology nursing research. While the emergence of big data and advances in analytic approaches provide new and exciting opportunities to advance oncology nursing science, they pose several challenges for nurse clinicians and researchers. For nurse clinicians, these challenges may include the facilitation of data collection from remote devices, staying current of rapidly evolving genomic tests to provide patient education and support,^87^ and translating findings from big data analyses into practice. Nurse researchers require education and training to develop research questions using and surmount challenges associated with rapidly evolving data analytic methods. For both nurse clinicians and researchers, ethical challenges associated with big data are ongoing and are likely to become more prominent with the increasingly ubiquitous nature of passive data collection. Common to each of these challenges is the need for education. As stated previously, all nurses need to understand big data, both its applications and limitations. Nursing programs need to provide courses on big data at all levels that include discussions of ethics and statistical methods. Nurses who are trained in big data and advanced computational methods will be poised to contribute to guidelines and policies that preserve the rights of human research participants. Big data has the potential to provide a current, comprehensive, and holistic representation of the patient’s experience. With their unique focus on the interplay between the whole person, the environment, and health, nurses bring an indispensable perspective to the interpretation and application of big data research findings. Using these approaches, oncology nurses will stay on the forefront of advancements in big data approaches and harness big data to improve the outcomes of patients with cancer.

## Figures and Tables

**TABLE 1:** Educational resources and training opportunities for nurses on use ofbig data.

Content Area	Name	Description	Location of Course/Training

Data Science Post-doctoral Fellowship	Big Data Scientist Training Enhancement Program	• Two-year fellowship offered through the National Cancer Institute and Veterans Health Administration of the USA • Goal is to leverage data science to advance cancer research through training, clinical guidance, and use of the VA health data infrastructure	Onsite at one of four VA medical centers Applicants must be a citizen of the USA; have proficiency in at least one programming language, and have experience in bioinformatics, modeling, or management of large datasets
• EMR • Statistical Methods	Electronic Medical Records Boot Camp: Biostatistical Methods for Analyzing EMR Data	• Two-day intensive boot camp featuring hands-on training and seminars • Provides an overview of electronic health data opportunities, statistical challenges, and latest techniques	Columbia University SHARP Training Program, New York, NY, USA Offered online
• Omics	Big Data Training for Cancer Research	• 10-day intensive workshop geared toward cancer researchers • Workshop covers managing, visualizing, analyzing, and integrating various types of omics data	Purdue University, West Lafayette, IN, USA Offered online or in-person
• Genomics	Computational Genomics	• Seven-day intensive course focused on the theory and practice of algorithms in computational biology • Course topics include statistical considerations in the design and analysis of genomic experiments	Cold Springs Harbor Laboratory Offered online
• Genomics	Quantitative Genomics Training: Methods and tools for whole-genome and transcriptome analyses	• Two-day intensive boot camp featuring hands-on training and seminars • Provides an overview of concepts, methods, and tools for whole-genome and transcriptome analyses in human health studies	Columbia University SHARP Training Program, New York, NY, USA Offered online
• Epigenetics	Epigenetics Boot Camp: Planning and Analyzing DNA Methylation Studies for Human Populations	• Two-day intensive boot camp featuring hands-on training and seminars • Provides overview of concepts, techniques, and data analysis methods utilized in human epigenetic studies with a focus on DNA methylation array	Columbia University SHARP Training Program, New York, NY, USA Offered online
• Microbiome	Microbiome Data Analytics Boot Camp: Planning, generating, and analyzing 16s rRNA gene sequencing surveys	• Two-day intensive boot camp featuring hands-on training and seminars • Provides an overview of 16s rRNA gene sequencing surveys including planning, generating, and analyzing sequencing datasets	Columbia University SHARP Training Program, New York, NY, USA Offered online
• Microbiome	Strategies and Techniques for Analyzing Microbial Population Structures (STAMPS)	• 10-day intensive course providing interdisciplinary training in bioinformatics and statistics • Topics include experimental design, assembly and annotation of shotgun metagenomic data, and statistical methods	Marine Biological Laboratory, Woods Hole, MA, USA Offered in-person
• Statistical methods • R programming • Omics	University of Washington Biostatistics Summer Institute in Statistical Genomics (SISG)	SISG is divided into several modules scheduled throughout summer • Provides instruction on the modern methods of statistical analysis	University of Washington, Seattle, WA, USA Offered in-person
• Statistical methods	University of Washington Summer Institute in Statistics for Big Data	• Four modules provided over 2 to 3 days • Introduces statistical techniques for the analysis of biological big data	University of Washington, Seattle, WA, USA Offered online
• Machine learning	Machine Learning Boot Camp: Analyzing Biomedical and Health Data	• Two-day intensive boot camp featuring hands-on training and seminars • Provides overview of statistical concepts, techniques, and data analysis methods for biomedical research	Columbia University SHARP Training Program, New York, NY, USA Offered in-person or online
• Machine learning	Introduction to Machine Learning for Epidemiologists	• Course is a month-long, 30-hour, self-paced digital course • Provides a broad exposure to machine learning and its practical applications within epidemiology	episummer, Columbia University, New York, NY, USA Offered online
• R programming • Python • Machine Learning • Text Mining	Bioinformatics Course Series and Workshops offered through FAES Academic Programs at the National Institutes of Health	• Courses are offered online in an asynchronous format • Workshops are offered online in a synchronous format	FAES Academic Programs, Bethesda, MD, USA Offered online
• R programming • Bioinformatics • Machine learning	Coursera (coursera.org)	• Self-paced online courses covering a variety of topics and levels of proficiency	Offered online
• R programming • Python • Machine learning	DataCamp (datacamp.com)	• Self-paced online courses covering a variety of topics and levels of proficiency	Offered online

Abbreviations: EMR = electronic medical record, FAES = Foundation for Advanced Education in the Sciences, SHARP = Skills for Health and Research Professionals, SISG = Summer Institute in Statistical Genomics, USA = United States of America, VA = Veterans Affairs
